# Csf1 Deficiency Dysregulates Glial Responses to Demyelination and Disturbs CNS White Matter Remyelination

**DOI:** 10.3390/cells9010099

**Published:** 2019-12-31

**Authors:** Bartosz Wylot, Jakub Mieczkowski, Sylwia Niedziolka, Bozena Kaminska, Malgorzata Zawadzka

**Affiliations:** Nencki Institute of Experimental Biology, 02-093 Warsaw, Poland

**Keywords:** microglia, Csf1, remyelination, osteopetrotic mice

## Abstract

Remyelination, a highly efficient central nervous system (CNS) regenerative process, is performed by oligodendrocyte progenitor cells (OPCs), which are recruited to the demyelination sites and differentiate into mature oligodendrocytes to form a new myelin sheath. Microglia, the specialized CNS-resident phagocytes, were shown to support remyelination through secretion of factors stimulating OPC recruitment and differentiation, and their pharmacological depletion impaired remyelination. Macrophage colony-stimulating factor (Csf1) has been implicated in the control of recruitment and polarization of microglia/macrophages in injury-induced CNS inflammation. However, it remains unclear how Csf1 regulates a glial inflammatory response to demyelination as well as axonal survival and new myelin formation. Here, we have investigated the effects of the inherent Csf1 deficiency in a murine model of remyelination. We showed that remyelination was severely impaired in Csf1-/- mutant mice despite the fact that reduction in monocyte/microglia accumulation affects neither the number of OPCs recruited to the demyelinating lesion nor their differentiation. We identified a specific inflammatory gene expression signature and found aberrant astrocyte activation in Csf1-/- mice. We conclude that Csf1-dependent microglia activity is essential for supporting the equilibrium between microglia and astrocyte pro-inflammatory vs. regenerative activation, demyelinated axons integration and, ultimately, reconstruction of damaged white matter.

## 1. Introduction

Inflammation, particularly manifested by microglia/macrophage activation contributing to antigen presentation and secretion of pro-inflammatory cytokines and toxic factors, has been long considered as detrimental to injured white matter in demyelinating conditions, such as multiple sclerosis (MS) [1,2,3,4,5,6,7,8,9,10]. Actually, post-mortem evidence [1,11,12,13] as well as experimental data [14,15,16,17,18] showed that remyelination occurs most efficiently in early active lesions characterized by the extensive activity of inflammatory cells. On the contrary, in chronic inactive MS lesions, which no longer contain inflammatory cells, oligodendrocyte progenitor cells (OPCs) fail to differentiate into mature cells and do not form new myelin [19]. It is well documented that endogenous myelin repair fails when the inflammatory response is experimentally reduced [20,21,22]. Moreover, the age-dependent decrease in remyelination is attributed equally to the intrinsic properties of aging OPCs [23] and the impaired response of aging microglia and macrophages to demyelination [24,25]. Both, activated microglia present in the CNS and macrophages derived from blood monocytes, contribute beneficially to remyelination as a source of growth factors during the early phases of regeneration, facilitating OPC recruitment [15] as well as phagocytose myelin debris that contain inhibitory factors deposits, which is required for oligodendrocyte differentiation and effective remyelination [26,27].

Differentiation of adult multipotent OPCs recruited to the demyelinated lesions is regulated by a specialized microenvironment shaped gradually during the resolution of the inflammatory process. It has been shown that in a response to the demyelinating insult, activated microglia initially polarize into a pro-inflammatory phenotype, exacerbating neurotoxicity, and then to an anti-inflammatory, pro-regenerative phenotype, conferring neuroprotection [28]. These polarized states represent the cells with antagonistic functions: the cells of enhanced antigen presentation properties, pro-inflammatory cytokine secretion, reactive oxygen and nitrogen species production, as well as promoting the Th1/Th17 adaptive immune response (traditionally classified as M1 cells) and the cells capable of phagocytosing tissue debris, promoting wound healing, inhibiting excessive inflammation, and suppressing adaptive immunity (M2 cells). Those states of microglia are controlled by specific factors produced within an affected tissue environment, such as pro-inflammatory cytokines, co-stimulatory molecules including CD80/86, effector enzymes such as iNOS, and NADPH oxidase or anti-inflammatory cytokines, immunosuppressive arginase 1 and scavenger receptors, respectively [29,30].

The M1/M2 transition is critically required for the restoration of tissue homeostasis and improvement of functional outcome. The pro-regenerative activated microglia and macrophages, the M2 cells, gradually increase in number during the process of inflammation, while the number of M1 cells is decreased. Finally, M2 microglia and macrophages are predominant in the injured white matter in the late phase of tissue response to the demyelinated insult and they mediate suppression of inflammation by releasing a variety of anti-inflammatory cytokines such as IL-4, IL-10, IL-13, IL-33, and TGF-β and/or support the regenerative reconstruction by producing growth and regulatory factors [31]. In addition, M2 microglia/macrophages promote the differentiation of Th2 and regulatory T cells, which in turn, contribute to suppression of inflammation by inhibition of Th1 cell activity, pro-inflammatory cytokine expression or secretion of growth regulatory proteins [32]. Moreover, the pro-regenerative activity of white matter microglia/macrophages promotes OPCs differentiation that leads to effective remyelination. M2 microglia-driven regenerative response has been proposed to be mediated, at least in part, by secretion of the TGFβ superfamily member activin-A, that directly drives OPCs differentiation [31]. The latest might be particularly important since inhibition of oligodendrocyte differentiation is a feature of chronic multiple sclerosis lesions [19].

Nevertheless, the recent data suggest that microglia rarely exist in stable phenotypes but flexibly respond to a specific combination of factors, actually present in the microenvironment [30]. The most potent factor controlling the development, survival, and local self-renewal of the microglial population as well as its regenerative polarization is colony stimulating factor 1 (Csf1), a key regulator of myeloid lineage cells [33]. Osteopetrotic mice are homozygous for a spontaneous recessive mutation in the Csf1 gene that causes Csf1 deficiency (Csf1-/-), which affects myeloid lineage development resulting in a significant reduction of monocyte/macrophage cell number in many tissues, including CNS [34,35]. We and others have previously shown significantly impaired accumulation of microglia/macrophages in CNS of Csf1-/- mice under various CNS pathological conditions, including demyelination [36,37,38,39].

Here, using a toxin-induced demyelination model in Csf1 deficient mice, we demonstrate that disruption of microglia function profoundly disturbs the white matter remyelination which is associated with alterations of pan-glial inflammatory responses. Strikingly, Csf1-mediated microglia accumulation and maturation is critical for maintaining a dynamic balance between microglia and astrocyte pro-inflammatory and regenerative responses. Thus, the temporal coordination of these phenotypes of both types of glial cells is required for axon survival, complete restoration of the myelin sheath, and ultimately the amelioration of white matter pathology.

## 2. Materials and Methods

### 2.1. Animals

All research and animal care procedures were approved by the First Warsaw Local Ethics Committee for Animal Experimentation (protocols 345/2012 and 797/2015) and performed according to international guidelines on the use of laboratory animals.

The animals were housed under standard laboratory conditions on a 12 h light/dark cycle with constant access to food and water and were randomly assigned to experimental groups. Nine to twelve-week-old female B6; C3Fe a/a-Csf1op/J mutant mice (The Jackson Laboratory, Bar Harbor, ME, USA, Stock No: 000231; Csf1-/-) and their wild type littermates were used. Breeding pairs of heterozygous mice were maintained in a barrier facility. Homozygous mutants were distinguished from wild-type and heterozygous mice at 10–11 days of age by the absence of incisors. For the breeding purpose, all mice were genotyped using the TaqMan allelic discrimination method of single base insertion within the coding region of Csf-1. The primer sequences were 5′-CAGGATGATCCTGTTTGCTACCTAA-3′ (forward) and 5′-GCGCATGGTCTCATCTATTATGTC-3′ (reverse); the probe sequences were VIC-AGGCCTTTTTT TCTGGTACA-MGB and 6-FAM-AAGGCCTTTTTT CTGGTACA-MGB. P0-P2 pubs of both, wild type and Csf1-/- were used for setting the astrocyte cultures. Transgenic eGFP-expressing mice C57BL/6-Tg (CAG-EGFP)1Osb/J (The Jackson Laboratory, stock number 003291, background C57BL6/J) were used as the donors of hematopoietic stem/progenitor cells. In heterozygotes, eGFP under the control of the chicken beta-actin promoter is widely expressed in all cells.

### 2.2. Surgical Procedures

Demyelination in mice was induced by injection of 1 µL of 1% L-α-lysophosphatidylcholine (lysolecithin, SIGMA, St. Louis, MI, USA) into the ventral and dorsal white matter funiculi at the level of T12 as previously described in detail [40,41]. Briefly, the position of T13 was identified and the epaxial musculature was cleared from the immediate area. The space between T12 and T13 was exposed and carefully cleared, the central vein was identified, and the dura was pierced with a dental needle lateral to the vein. A three-way manipulator was then used to position the needle for stereotaxic injection of EB. Hamilton needle with a fine glass tip was advanced through the pierced dura at an angle appropriate for ventrolateral or dorsal funiculus injection. The injection was controlled at 1 µL per minute and the needle remained in the injection site for 2 min to allow maximal diffusion of toxin.

During all surgical procedures, animals were anesthetized with isoflurane supplemented with buprenorphine (0.03 mg/kg, intraperitoneal injection) for pain relief.

### 2.3. Tissue Processing

For fresh-frozen tissue isolation, animals were terminally anesthetized with isoflurane at 6 dpl (n = 3) for each experimental condition and perfused through the ascending aorta with sterile, ice-cold PBS. The spinal cord chunks containing lesions were immediately removed and frozen in isopentan alcohol. The spinal cord chunks containing lesions were cryosectioned into 20 μm slices and mounted on PEN membrane (polyethylene naphthalate, Arcturus, ThermoFisher Scientific, Waltham, MA, USA) for further laser captured microdissection. For in situ hybridization and immunodetection, animals (n = 5 for each experimental condition) were terminally anesthetized with pentobarbitone and intracardially perfused with 4% (*w*/*v*) paraformaldehyde (PFA, Sigma, St. Louis, MI, USA) in phosphate buffered saline (PBS, pH 7.4) at the indicated time after surgical procedure. Lesion containing tissue was dissected, post-fixed in 4% PFA overnight then immersed in 30% sucrose solution prepared with PBS for 48 h before embedding with OCT (ThermoFisher Scientific, Waltham, MA, USA). Twelve micrometer sections were thaw-mounted onto poly-l-lysine coated slides and stored at −80 °C until further use. Some animals were perfused with 4% glutaraldehyde for electron microscopy.

### 2.4. Immunofluorescence

Twelve micrometer cryo-sections were blocked with 10% donkey serum in PBS for 2 h at room temperature, incubated for 12 h at 4 °C with primary antibodies followed by incubation with fluorescent dye-conjugated secondary antibodies for 2 h at RT. The following primary antibodies were used: Iba1 (rabbit, 1:1000, Wako Chemicals, Richmond, VA, USA), IB4 (Sigma), Olig2 (rabbit, 1:500, Millipore, Burlington, USA), GFAP (rabbit, 1:1000, DAKO, Glostrup, Denmark), CC1 (mouse, 1:200, Calbiochem, Merk, Darmstadt, Germany), SMI32 (Sternberger Monoclonals Inc., Lutherville, MD, USA), MBP (mouse, 1:200, R&D Systems, Minneapolis, MN, USA), AGR-1 (goat, 1:100, Santa Cruz, CA, USA), iNOS (rabbit, 1:100, Millipore). Then, sections were incubated with appropriate AF488, AF555, or AT647- conjugated secondary antibodies (1:500, ThermoFisher Scientific). Nuclei were visualized with 4′,6′-diamidino-2-phenylindole (DAPI; 0.1 mg/mL; Sigma). Images were acquired with fluorescence (Leica DM4000B) and confocal (Zeiss LSM700, Carl Zeiss, Oberkochen, Germany) microscopes. Images were analyzed and staining intensity was quantified with ImageJ software (https://imagej.nih.gov/ij/, NIH, USA)

### 2.5. Oli-Red-O Staining

Oil-red-O (Sigma) working solution was prepared by adding 20 mL H_2_O to 30 mL 1% oil-red-O in isopropanol. Sections were stained for 10 min then washed for 4 min and counterstained in hematoxylin for 1 min. Following a 5-min wash in water, the sections were differentiated in 0.5% aqueous hydrochloric acid and again washed in water for 10 min. Finally, the slides were mounted using an aqueous mounting medium. Representative images of oil-red-O stained lesions were digitized and densitometrically assessed.

### 2.6. Semi-Thin Resin Sections, Rank Analysis, and Electron Microscopy Analysis

Tissue was post-fixed in glutaraldehyde solution and then cut transversely into 1.0 mm-thick blocks. Blocks were further fixed in osmium tetroxide, dehydrated through ascending ethanol washes and embedded in TAAB resin.

Semi-thin resin sections (six lesions per group, two sections per lesion) of the toluidine blue-stained spinal cords were analyzed to assess normal myelin, demyelinated axons, and extend of remyelination. Remyelinated axons were distinguished from normally myelinated axons outside of the lesion by the thinness of the myelin sheath. Within the lesion, demyelinated axons were identified by the absence of a myelin sheath while remyelinated axons possess myelin sheaths with a dark staining rim and that are thinner than would be expected for the axonal diameter. Using these morphological criteria, the estimated degree of remyelination was ranked under blind conditions. Statistical analysis on the rank scores was performed using Mann–Whitney test. Photographs were taken under a Leica microscope (Leica, DM4000B, Wetzlar, Germany) and digital camera. For ultrastructural analysis, selected blocks of resin-embedded tissue were trimmed, and ultrathin sections (50–70 nm) were cut onto copper grids. The tissue was stained with lead citrate and uranyl acetate according to standard protocols before being examined by transmission electron microscope JEM 1400 (JEOL Co., Tokyo, Japan).

### 2.7. In Vitro Cell Culture and Treatment

Primary mixed glial cultures were prepared from P0-P2 wild type C57BL6/J or mutant Csf1-/-mice as previously described [42]. Briefly, meninges were removed from brain hemispheres, forebrains were enzymatically (0.025% trypsin at 37 °C for 20 min) and mechanically dissociated to a single cell suspension, and cells were plated at a density of 3 × 10^5^ cells/cm^2^ on poly-L-lysine-coated flasks in Dulbecco’s modified Eagle medium (with Glutamax, 4.5 g/L glucose, 10% heat-inactivated fetal bovine serum FBS, Gibco), 100 U/mL penicillin, and 0.1 mg/mL streptomycin). After 8 days, microglial cells were removed by mild shaking over 1 h and then cultures were shaken for an additional 16 h on a rotary shaker at 37 °C at 250 rpm to detach OPCs. Remaining adherent astrocytes were re-seeded on poly-l-lysine-coated culture plates at a concentration of 50 cells/mm^2^ and maintained for 48 h before treatment in DMEM containing 10% fetal calf serum (with 2 mM glutamine and antibiotics). Lineage negative hematopoietic progenitor cells were obtained from GFP-expressing mice and added onto the confluent astrocyte monolayer. Then, cells were cultured with a culture medium in the presence or absence of Csf2 (10 ng⁄mL; Peprotech, London, UK) for 7 DIV.

### 2.8. Hematopoietic Precursor Isolation

The multipotent hematopoietic stem/progenitor cells with a classic signature of LSK phenotype (lineage negative, Sca-1+, cKit+, HSCs/HPCs [43]) were purified from the bone marrow (BM) of adult GFP-positive mice using antibodies recognizing cKit and Sca1 cell surface proteins, specific for hematopoietic precursors and lineage differentiation markers, typically present on mature blood cells. Bone marrow cells were collected from 8- to 10-week-old mice by flushing out the femora and tibiae of the hind limb under deep anesthesia. Erythrocyte removal was performed by lysis with 1 × BD Pharm Lyse Buffer (BD Biosciences, San Jose, CA, USA) and cells were washed twice in phosphate-buffered saline. A single-cell suspension of total nucleated cells was incubated at 4 °C for 30 min with a set of rat monoclonal antibodies against mice lineage markers CD3, CD4, CD5, CD8a, CD11b/MAC-1a, B220, Gr-1, and TER-119 (R&D Systems); CD45 and antibodies against stem cell antigens CD133, c-kit. Cells were washed and re-suspended in cold buffer and sorted with FACS Aria II sorter using FACS Diva software (v8.0, BD Biosciences, San Jose, CA, USA) to obtain cell population enriched in HSPCs (Lin-Sca1+CD45+CD133+; for FACS sorting gating strategy see Figure 4A). Cell aggregates were removed from analysis during each experiment by doublet exclusion using pulse processing-based FSC-H vs. FSC-W method. For clonogenic evaluation, potential cells were plated in methylcellulose to grow hematopoietic colonies. HSPCs were plated over astrocyte monolayer in α-minimum essential medium with 20% fetal bovine serum (GIBCO) at the density of 1 × 10^4^ per 24-wells plate for 7 days and subsequently washed and fixed with 4% of paraformaldehyde.

### 2.9. Global Gene Expression Analysis

White matter lesions were visualized in tissue sections by staining with hematoxylin to visualize tissue cellularity on ice for 5 min and immediately dehydrated. All solutions were prepared using RNAse-free water. Demyelinated lesions, defined by high cellularity within white matter, were dissected form each section through the whole lesion volume with Laser Capture Microdissection System PixCell II (Arcturus, ThermoFisher Scientific). Lesions were cut out using UV laser and capture with IR laser as quickly as less than 2 h for one slide. Dissected fragments obtained from each animal were pulled and then RNA was extracted using PicoPure RNA Isolation Kit (ThermoFisher Scientific). The quality and integrity of the RNA was determined using the Agilent 6000 RNA Nano kit and an Agilent Bioanalyzer 2100 (Agilent, Santa Clara, CA, USA). Fragmented cDNA was amplified and biotin labelled employing the 3′IVT express kit (Illumina) and hybridized to the whole-genome gene expression microarrays (Illumina Mouse WG6v2) with five biological replicates per condition.

The microarray experiments were conducted at the microarray facility of the Cambridge Genomic Service, Cambridge, UK according to standard protocols. The microarray dataset has been deposited in the GEO repository, and is available with the accession number GSE93645.

### 2.10. Statistical Analysis of Microarray Data

Microarray data were exported from Illumina BeadStudio software (San Diego, CA, USA), processed and normalized using the R/Bioconductor beadarray, lumi, and limma packages. Before normalization, probes that were not detected (detection *p* value > 0.01) were removed from further analysis. Remaining probes were mapped to gene identifiers from Ensembl database (gene_stable_id). For each gene, we computed a single average intensity profile from the profiles of all the probes mapped to it. The resulting average profile was then log2-transformed and used in statistical analysis and visualization. Differential expression analyses were conducted using the limma Bioconductor package (3.0, San Francisco, CA, USA). False discovery rate (FDR) was used to adjust for multiple hypotheses testing [44]. Genes with FDR < 0.05 and with at least 1.5-fold change in expression levels were found as differentially expressed. Signalling pathways described in the Gene Ontology (GO) resource were tested for overrepresentation in the list of differentially expressed genes using Fisher’s exact test.

### 2.11. Quantification and Statistical Analysis

Data analysis was performed using Prism 6 (GraphPad, San Diego, CA, USA) software except for microarray analysis. Data are represented as mean ± sd. To detect differences between experimental conditions Student’s t-test was performed. For all tests, *p* = 0.05 was taken as the minimum level of statistical significance (* *p* < 0.05; ** *p* < 0.01; *** *p* < 0.001). In vitro assays represent three independent experiments from individual culture preparations.

## 3. Results

### 3.1. Csf1 Deficiency Disturbs Remyelination despite Unaltered Recruitment and Differentiation of OPCs

**Figure 1:**
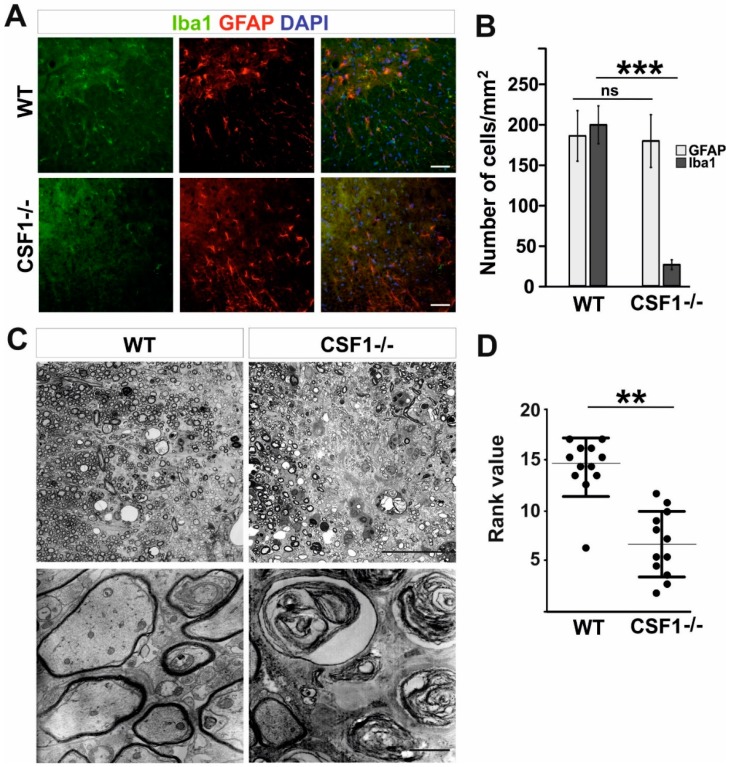
Csf1 deficiency results in severe remyelination failure. (**A**). Severe depletion of microglia but not astrocytes in transverse 12 µm sections of spinal cord white matter of 8–10 week old WT and Csf1-/- mice immunostained with anti-Iba1 antibody and GFAP. DAPI (4′,6′-diamidino-2-phenylindole) shows nuclei counterstaining. Scale bar represents 50 µm. (**B**). Quantification reveals significant reduction in Iba1+ cell number in spinal cord white matter of Csf1-/- mice compared to WT mice (N = 3 per genotype) while GFAP+ cell number was unaffected. Data shown as mean ± SD. Statistical significance was determined by Student’s t test with *** *p* < 0.001. (**C**). Microscope images of semi-thin sections stained with toluidine blue and electron micrographs of ultrathin sections from control (**left panel**) and Csf1-/- (**right panel**) lesioned mice 28 days after demyelination (scale bar = 50 µm in upper images and 500 µm in lower images). Rank analysis of remyelinated lesions is shown in (**D**). Mann–Whitney test, ** *p* < 0.01.

First, we confirmed a significant reduction in the number of microglia occupying intact spinal cord white matter of Csf1-/- mutant mice compared to their wild-type littermates. Expression of ionized calcium binding adaptor molecule 1 (Iba1), which sensitively marks microglia, revealed the significantly decreased number of Iba1 positive cells in Csf1-/- mice compared with their wild-type littermates (25.8 ± 5.9 vs. 176.0 ± 32.0 cells/mm^2^, respectively; mean ± SD, *p* < 0.001, approx. 85% reduction in the number of cells). We found no difference in the number of white matter astrocytes between mutants and wild types (195.6 ± 22.9 vs. 182.1 ± 30.6 cells/mm^2^; Figure 1A,B).

No apparent difference in microglia morphology between wild-type and mutant mice was observed (Figure 1A). Accordingly, the Csf1-/- mice represent a model of profound microglia deficiency in a spinal cord white matter and as such are useful to investigate the role of microglia depletion and Csf1-Csf1R signalling-mediated mechanism in the inflammatory phase of remyelination. To this end we used a well-described model of demyelination/remyelination employing stereotaxic injection of membrane solubilizing agent, lysolecithin, into the white matter of murine spinal cord that allows investigating the whole process through its well-defined kinetics and critical stages [40,41,45]. Relatively large demyelinating lesions are observed in spinal cord white matter as soon as three days after toxin injection and they reach their maximum at 6 dpl.

To examine whether Csf1 deficiency influenced the effectiveness of remyelination, mice were injected with 1 µL of 1% lysolecithin into the ventral and dorsal funiculi of the spinal cord to induce focal demyelination. Toluidine blue staining of semi-thin resin sections revealed impaired remyelination in Csf1-/- mice with the presence of extensive non-remyelinated areas within the lesion at 28 dpl (Figure 1C) as assessed by the standard ranking analysis (Figure 1D). Extensive axonal degeneration was evident in electron microscopy images (Figure 1C, lower panel). Such axonal abnormalities were not found in the white matter of the control WT animals which showed, as expected, complete remyelination.

**Figure 2:**
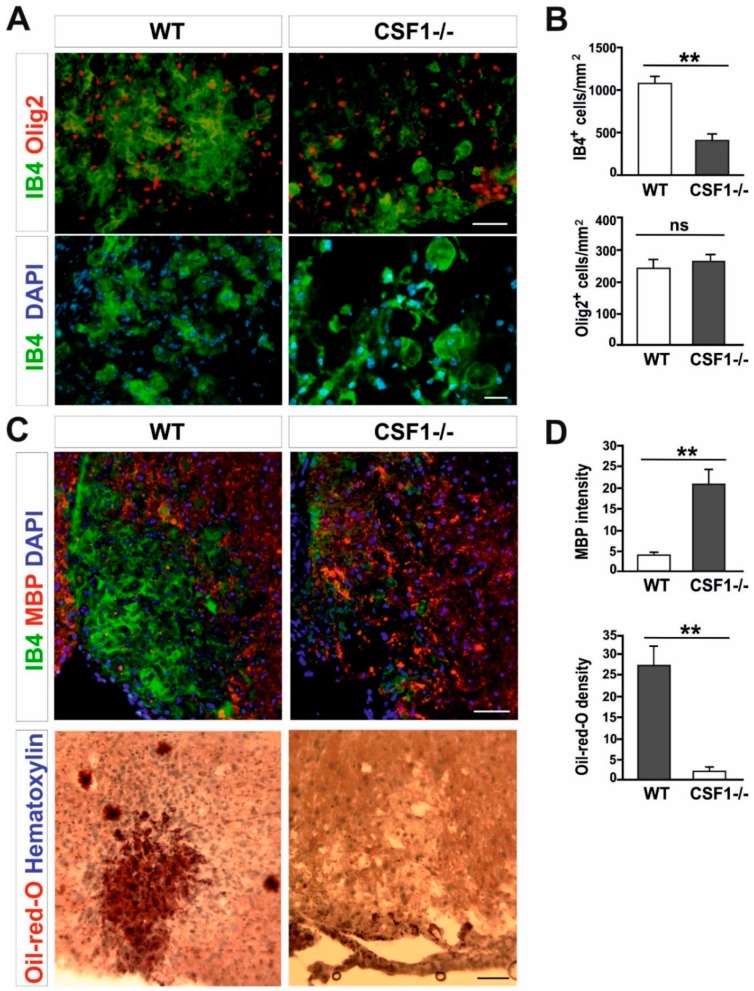
Csf1 deficiency disturbs microglia proliferation and function but not OPCs recruitment to the lesion. (**A**). Transverse sections of spinal cord white matter lesions stained with IB4 for activated microglia/macrophages and Olig2 show reduced number (upper panel, scale bar 50 µm) and different morphology (lower panel, scale bar 20 µm) of IB4-labelled cells but unaltered number of OPCs in Csf1-/- mice at 10 dpl as quantified in (**B**). Lysolecithin was injected unilaterally into the ventral funiculus of control and Csf1-/- mice to induce focal demyelination (N = 4–5 per genotype). (**C**). Transverse section of ventral funiculus lesions co-immunostained with IB4 and myelin basic protein (MBP) (upper panel) and Oil-red-O (lower panel) to allow identification of the extent of phagocytosis. Immunostaining reveals accumulation of undigested MBP and significantly less intracellular lipids at day 10 post lesion in Csf1-/- animals than in control animals, indicating that phagocytic activity is defective (scale bar 50 µm) as quantified in (**D**). The quantification of myelin debris in demyelinated lesion areas was performed by histological analysis of MBP intensity with ImageJ software. Data shown as mean ± SD. Statistical significance was determined by Student’s t test with ** *p* < 0.01.

Next, we investigated a number of microglia/macrophages recruited to the lesion areas in Csf1-/- mice after 10 days post-lesion, given that there is a maximum of cell proliferation and migration between 6 and 10 dpl in wild-type controls [40]. We found substantial reduction of microglia/macrophage accumulation identified as IB4 lectin binding cells in the demyelinating lesion of Csf1-deficient mice; significantly fewer IB4+ cells were present within lesions of mutant mice than in wild-type controls (406.8 ± 176.9 vs. 1077.8 ± 187.4 cells/mm2, respectively; mean ± SD, *p* < 0.001; Figure 2A,B).

Possible explanations of the remyelination failure include defects in OPCs recruitment to the demyelination area or inhibition of their differentiation into myelinating oligodendrocytes, usually associated with ineffective clearance of myelin debris by macrophages. To verify the first of these hypotheses we assessed the number of OPCs occupying the demyelinated lesion at 10 dpl, when OPCs reach the maximum of their migration and proliferation in wild-types. Interestingly, recruitment and proliferation of OPCs in the lesions of mutant mice appeared not affected; we observed a similar number of Olig2+ cells within lesions of mutant and controls (Figure 2A,B). This suggests that the limited number of activated microglia could be sufficient to trigger OPCs activation, recruitment, and proliferation within the injured white matter.

Since the number of OPCs was the same in both Csf1-/- and control animals, we hypothesized that impaired remyelination might be a consequence of limited phagocytosis of myelin debris. Therefore, we used two indicators to assess the efficiency of myelin debris phagocytosis: immunostaining for myelin basic protein (MBP) which is an important structural protein of myelin sheaths and Oil-red-O staining that allows identification of neutral lipids that are formed within the white matter only when myelin debris has been ingested by macrophages [27]. Accordingly, in the white matter of Csf1-/- lesions, we found an abundant accumulation of non-structural myelin fragments, as detected by the localization of MBP immunoreactivity outside of macrophage cells, which was not present in wild type lesions at the same time after demyelination. This accumulation was accompanied by virtually none Oil-red-O-stained macrophages, in contrast to control animals, indicating that the Csf1 depletion results in the inhibition of myelin debris clearance (Figure 2C,D).

**Figure 3:**
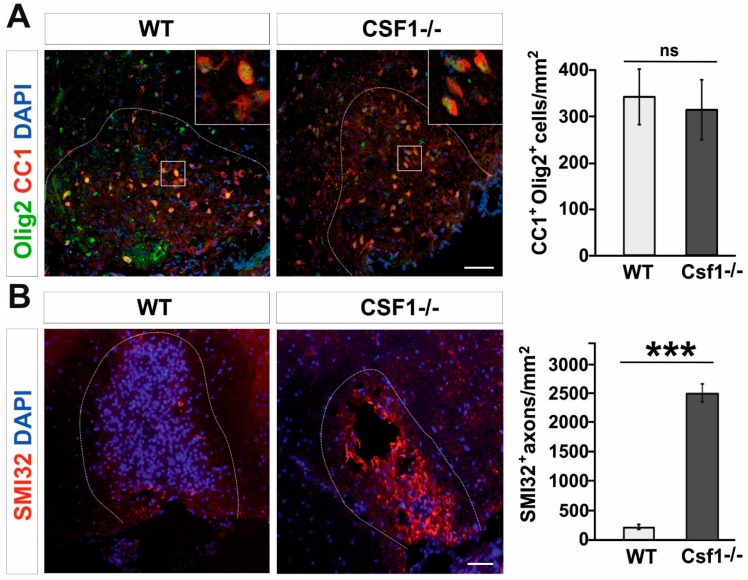
Oligodendrocyte differentiation is not altered while axons are dephosphorylated in Csf1-/-lesions. (**A**). Representative confocal images showing CC1/Olig2-positive differentiated oligodendrocytes in Csf1-/- and WT lesions at 28dpl. Dashed lines denote demyelinating lesion area. Scale bar = 50 μm. High confocal power images (to the right) show the details of boxed areas. Cell density quantifications reveal no differences between WT and mutant mice as shown in the graph. Data are shown as mean +/- SD. (**B**). Dephosphorylated NFs of damaged axons detected by the SMI32 antibody in the demyelinating lesion of Csf1-/- mice. Mean ± SD, *** *p* < 0.001.

However, while examining the number of mature oligodendrocytes present in lesions at 21 dpl, when remyelination is usually completed in wild type animals, we found that CC1+/Olig2+ double positive oligodendrocytes populated the lesion area with similar density in both Csf1-/- mutants and WT (Figure 3A). This implies that there is no direct link between the OPC differentiation and the efficiency of myelin debris clearance in mutant mice. Our results show that neither OPC proliferation nor their differentiation was significantly impaired in Csf1-/-.

Myelination induces and controls phosphorylation of axonal neurofilaments (NFs) that correlates with fast axonal transport [46,47]. Non-phosphorylated NFs are predominantly found in damaged axons and lack of proper phosphorylation of NF may contribute to further axonal pathology [48]. Dephosphorylated neurofilaments can be easily visualized in spinal cord sections since dephosphorization uncovers epitopes particularly accessible to SMI32 antibody, which are otherwise hidden [49]. Since neurofilaments in healthy myelinated axons are heavily phosphorylated and therefore stay SMI32-negative, SMI32 immunoreactivity provides a sensitive marker for demyelination and axonal pathologic changes during neurodegeneration [50], ALS [51] and MS [52,53]. We found that in Csf1-/- mice lesions at 10 days after demyelination contain abundant dephosphorylated, SMI32–positive axons, while in wild type lesions axons were not affected (Figure 3B).

### 3.2. Csf1 and Csf2 Differentially Alter the Proliferation and Maturation of Monocyte-Lineage Progenitor Cells In Vitro

**Figure 4:**
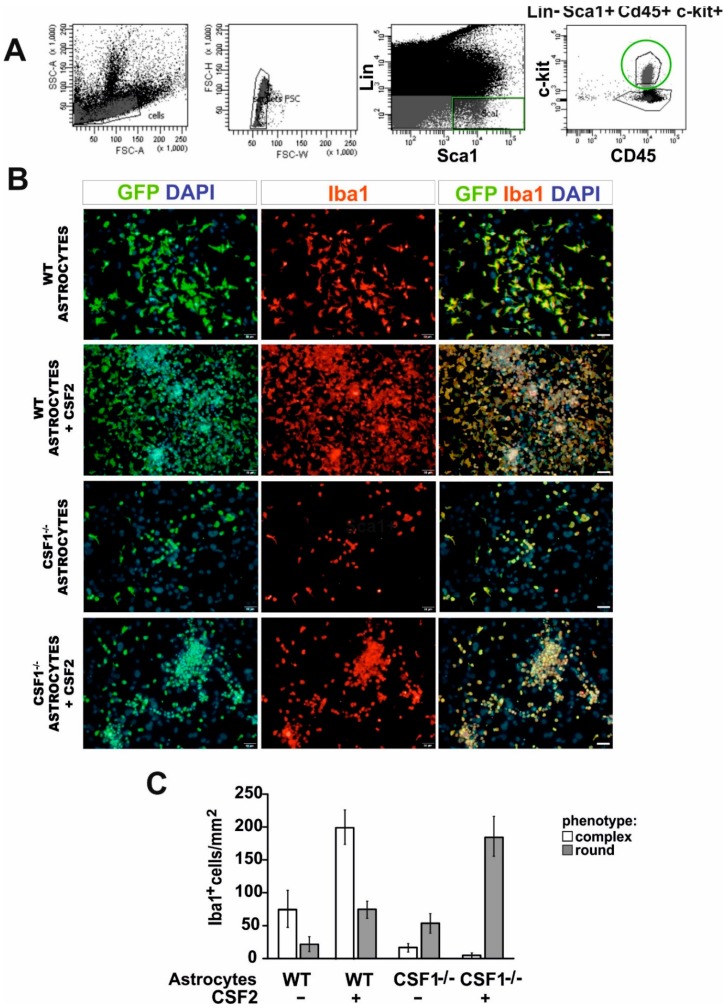
Differentiation of LSK cells into microglia-like cells in vitro is differentially dependent on Csf1 and Csf2. (**A**). FACS sorting strategy for isolation of the bone marrow-derived hematopoietic stem/progenitor cells of CD45^+^lin^−^Sca1^+^c-kit^+^ phenotype (LSK) from GFP-positive mice. (**B**). Sorted cells were co-cultured with primary astrocytes from wild type and Csf1-deficient mice with or without the addition of Csf2 (10 ng/mL) for 7 days and subsequently stained with anti-Iba1 antibody. (**C**). Quantification of the effect of Csf1 and Csf2 on LSK cells differentiation/maturation. Two phenotypes observed among GFP+ cells were quantified in at least three different microscope fields. Data are presented as mean ± SD.

Next, we sought to define the identity and origin of myeloid lineage cells reacting to demyelination in Csf1-/- mice. Therefore, we examined whether the bone marrow-derived hematopoietic stem/progenitor cells of the CD45^+^lin^−^Sca1^+^c-kit^+^ phenotype (HSPCs, LSK signature) could be a source of functional macrophages in the injured CNS in a context of Csf1-deficiency. To specify the role of Csf1 in HSPCs proliferation, differentiation, and monocyte maturation, we performed in vitro test allowing for differentiation of progenitor cells in close contact with pure astrocyte cultures derived from Csf1-/- and wild type mice. HSPCs were isolated from the bone marrow of GFP-positive mice by single-cell FACS (a FACS gating strategy is presented in the Figure 4A), individual colonies were expanded in vitro and then subjected to co-culture with astrocytes for 7 days. The effect of Csf1 and Csf2 on cell differentiation/maturation was examined by co-culture of the GFP+ HSPCs with astrocytes derived from wild type and Csf1-deficient mice with or without the addition of Csf2 (10 ng/mL). CD45^+^lin^−^Sca1^+^c-kit^+^ cells differentiated into Iba1-positive macrophage-like cells when co-cultured with wild type astrocytes. Csf1-deficiency resulted in the presence of a low number of round-shaped Iba1-positive cells in the co-cultures. In contrast, massive proliferation of CD45^+^lin^−^Sca1^+^c-kit^+^-derived cells after additional treatment with Csf2 was observed regardless Csf1 presence or absence. Cell proliferation was severely reduced in Csf1 deficiency conditions. Importantly, we found that morphology of cells cultured over WT astrocytes was complex, while cells cultured with Csf1-/- astrocytes showed a uniform round shape phenotype (Figure 4B,C).

### 3.3. Reduction of Microglia/Macrophages Alters Pan-Glia Response Dynamic after Demyelination

**Figure 5:**
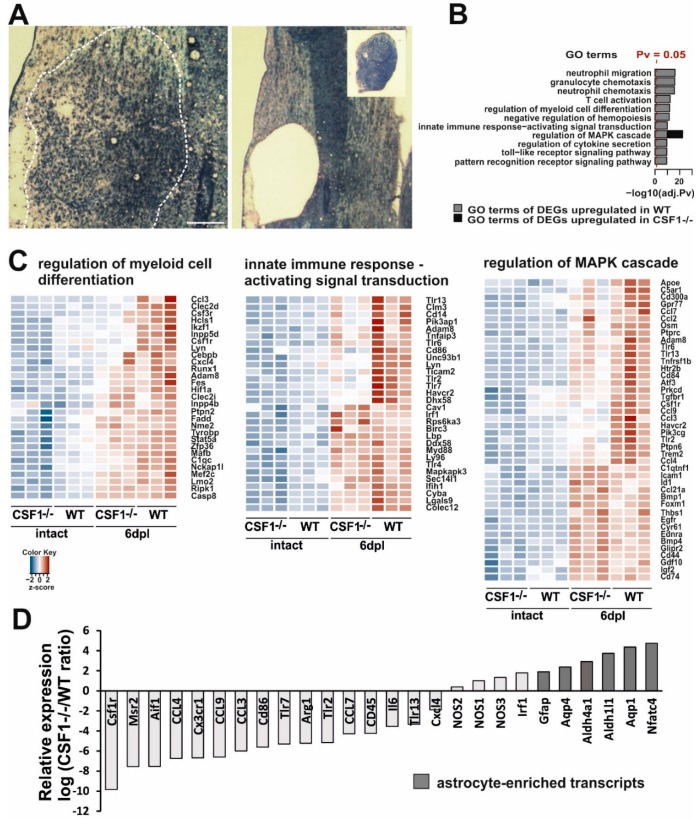
Transcriptional analysis of demyelinated white matter in Csf1-deficiency condition. (**A**). Representative images of demyelinating lesion at 6 dpl, note the maximum level of cell migration and activation as evidenced by detection of increase of tissue cellularity (**left**). The lesion area depicted were microdissected with IR and UV lasers for gene expression analysis (**inset**, **right**). (**B)**. Top enriched Gene Ontology “biological process” terms of significantly most upregulated genes at 6 dpl in wild types and Csf1-/- mutants. Functional analysis of the transcriptome data comparing data from demyelinated white matter of WT and Csf1-/- vs. intact tissue were performed. Pathways with an adj. *p* value < 0.05 were considered significantly enriched and plotted with a - log 10 transformed adj. *p* value in descending order of statistical significance. Only the pathways with the highest enrichment were reported. Significantly enriched pathways in WT (blue bars), and Csf1(-/-) (red bar) were shown. (**C**). Unsupervised hierarchical clustering analysis using the differentially expressed genes (DEGs). Heatmap of selected enriched terms from GO pathway analysis of the most upregulated DEGs with an adjusted *p* value of less than 0.05 and log2 fold change of greater than 1.5 Genes are clustered together based on expression similarity. Heatmap colors correspond to gene expression as indicated in the color key: red (over-expressed) and blue (down-regulated). The color key scale bar at lower left shows Z-score values for the heatmap. (**D**). Microarray analysis restricted to the most enriched transcripts shows the distinct profiles in Csf1-/- and WT at 6 dpl after demyelination. For each individual gene a ratio between average expression in CsF1-/- and WT demyelinated white matter were computed. Expression ratios were plotted on a log2 scale. On the left are genes overexpressed in WT, and on the right genes overexpressed in Csf1-/- mice. Note a higher representation of astrocyte-enriched genes in Csf1-/- mice. Gene signatures for micro- and astroglial-lineage cells were extracted from the literature and crossed with our microarray data.

In a view of above, we assumed that the early innate immune response triggered by demyelination in Csf1-/- mice was limited only to the reaction of very low number of resident microglia. Therefore, we attempted to address how the reduction of microglia cell number as well as their proliferation and polarization ability affects post-injury inflammatory profile within demyelinated white matter. To this end we compared the global gene expression in the lesion area of Csf1-/- mice and their wild type littermates at 6 days after induction of demyelination. Although the microglial activation begins shortly after toxin injection, the maximal level of microglial activation, as evidenced by dramatic cellularity changes, is reached around 6 dpl (maximum increase of tissue cellularity as shown in Figure 5A).

Analysis of transcriptome within the lesion area revealed distinct transcriptional programs in the injured white matter of Csf1-/- and wild type mice (Figure 5). Given the well-recognized rapid response of microglial cells to traumatic stimuli, we expected a significant increase in the expression level of inflammatory-associated genes after demyelination in wild types. Actually, gene ontology analyses demonstrated that several functional categories from the GO: biological process ontology, namely: “neutrophil migration”, “granulocyte/neutrophil chemotaxis”, “T cell activation,” as well as “regulation of myeloid cell differentiation” were most strongly upregulated by the induction of demyelination as compared to intact white matter (Figure 5B). We found also that genes associated with signal transduction of the innate immune-activation including mitogen-activated protein kinase (MAPK) cascade and pattern recognition receptor signalling pathway (including Toll-like receptors TLR signalling) were significantly upregulated in wild type mice during the early response to demyelination.

For comparison of the sets of genes regulated in mutants and wild type mice, we choose a uniform *p*-value threshold of 0.05 in order to achieve gene sets of comparable sizes. After the filtration of the signals below the threshold, we performed an unsupervised hierarchical clustering analysis using the differentially expressed genes from selected categories showing relative changes in gene expression plotted against each other on the log2 scale (Figure 5C). Heatmap plots of differentially expressed genes across all samples demonstrated genes with at least a 1.5 log2 fold changes. We found an increase in the expression of genes associated with the regulation of myeloid cell differentiation—*Ccl3*, *Csf3r*, *Csf1r*, and *Cxcl4*. There was also the increased expression of genes encoding members of signal transduction pathways regulating innate immune responses, such as *Tlr3*, *Tlr6*, *Lyn*, *Tlr2*, and *Tlr7*. In contrast, the expression of genes associated with these functional categories was mostly highly downregulated in intact Csf1-/- mice and significantly less upregulated after demyelination.

The expression data showed that several genes from the category “regulation of MAPK cascade” were differentially regulated in both Csf1-/- and wild type mice (Figure 5B,C). Many of the genes within this category (*Apoe*, *Csf1R*, *Ccl7*, *Ccl2*, *Tlr6*, *Tlr13*, *Ccl9*, *Ccl3*, and *Clec7a*) are known to be developmentally and/or pathologically-regulated in microglia [54,55,56,57] and, as expected, were significantly less upregulated after demyelination in Csf1-/- than in wild types. Interestingly, within this category, we identified a group of genes significantly upregulated (*p* < 0.05) in Csf1-/- mice. These genes have been shown to be expressed specifically in astrocytes (*Id1*, *Bmp1*, *Bmp4*, *Cd44*, *Gdf10*, *Igf2*, and *Cd74* [58]) or oligodendrocyte progenitors (*Bmp4* [59]) after CNS white matter demyelination. Moreover, when comparing differences in transcriptome exclusively between demyelinated white matter of mutants and wild types at 6 dpl, we found that lesions in wild type mice were particularly enriched in transcripts strongly associated with the microglial expression signature (*Csf1R*, *Msr2*, and *Aif1*). In contrast, Csf1-/- lesions were enriched in several well-known astrocyte transcripts (*Gfap*, *Aqp4*, and *Aldh1l1*, Figure 5D). This suggests that some cells, most likely astrocytes, might have developed the aberrant inflammatory reaction in microglia-depleted white matter.

Interestingly, we found that expression of genes that have been proposed to be critically involved in M1/M2 switch, such as iNOS and Arg1, was significantly dysregulated in Csf1-/- as early as at 6 dpl. As soon as 6 days post-lesion, we detected significantly lower *Arg1* expression, while higher expression of genes encoding for three types of nitric oxide synthases (*NOS1*, *NOS2*, and *NOS3*) was found in Csf1-/- mice compared to wild types (Figure 5D). These results suggested dysregulation of innate immune responses and prompted us to examine the context-dependent profile of iNOS and Arg1 production within the injured tissue. We further characterized the cellular source of iNOS and Arg1 during the classical M1/M2 transition period (6–10 dpl), corresponding to the beginning of differentiation of OPCs that have been recruited into the lesion.

**Figure 6:**
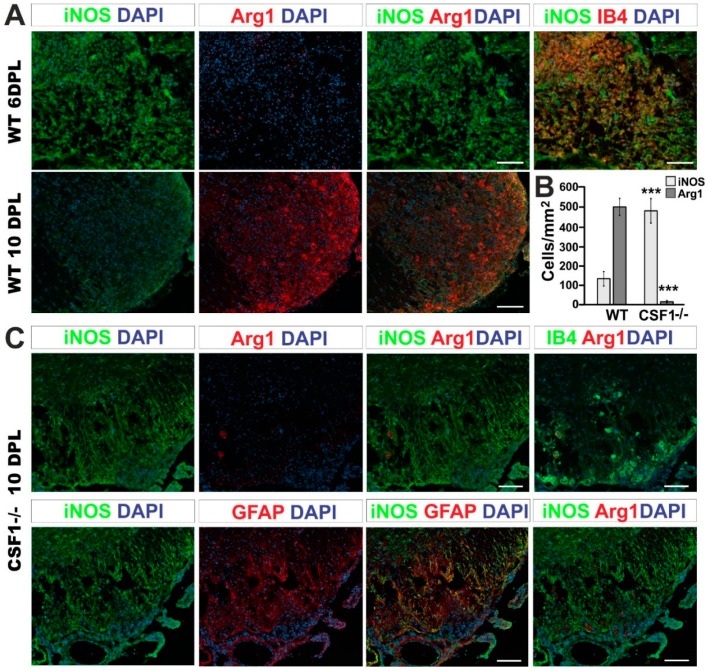
Csf1 deficiency results in a strong disequilibrium of microglia/astrocyte inflammation resolution. (**A**). Representative confocal images showing inflammatory/regenerative microglia phenotype switch identified by co-labelling of Arg1 with iNOS at 6 and 10 dpl focal demyelination in WT mice. Scale bar = 20 µm. (**B**). Quantification of iNOS and Arg1 expressing cells in the lesions of Csf1-/- and WT at 10 dpl. The cells were counted using ImageJ™ software from the 3–4 independent fields taken from each animal (N = 3–4 per group). Scale bar: 100 μm., mean ± SD, ***p < 0.001. (**C**). Representative fluorescence images of Arg1 and iNOS staining within the Csf1-/- lesions at 10 dpl show the localization of numerous iNOS-positive expressing cells in the lesion, in contrast to wild types shown in (**A**). Only very few of activated IB4-positive macrophages express Arg1 in the demyelinated white matter of Csf1-/- (**upper panel**). iNOS-producing cells have been identified as GFAP-positive astrocytes (**lower panel**). GFAP-positive cells do not express Arg1.

We performed immunohistochemistry using the antibody against Iba1 or IB4 lectin binding simultaneously with iNOS or Arg1 detection on sections from control and Csf1-/-mice. Accordingly, our immunofluorescent staining confirmed previously described kinetics of iNOS and Arg1 expression after demyelination in wild type mice [31]. We found that activated Iba1 positive cells were the main source of highly expressed iNOS at 6 dpl (Figure 6A, upper panel), while the Arg1 expression was low. Significant increase in Arg1 immunoreactivity associated with microglia/macrophages could be observed at 10 dpl in wild type mice (Figure 6A, lower panel, Figure 6B).

The immunoreactivity profile of both factors was dramatically different at 10 dpl after spinal cord demyelination in Csf1-/- mice. The population of iNOS-positive cells (regardless of their lineage identity) was 3.6 times more numerous in Csf1-/- mice than in WT mice. Moreover, only 1.2% of Arg1 expressing cells was observed in demyelination lesions of Csf1-/- mice in comparison to control mice (Figure 6B). Microglia in mutant mice acquired different morphology than cells activated within lesions in WT mice (Figure 6C). Only scarce microglia with a rounded, amoeboid morphology were found in location confined to the injured white matter. In contrast to the controls, in Csf1-/- spinal cord lesions, we found a large population of iNOS-positive cells as late as at 10 dpl (Figure 6B). The vast majority of iNOS-producing cells were GFAP-positive which implies a stronger pro-inflammatory response in astrocytes in Csf1-deficient mice (Figure 6C).

These data show that CFS1 deficiency affects the equilibrium between responses of microglia/macrophages and astrocytes and triggers important changes in a functional phenotype of these cells. This could be because the small number of malfunctioning microglia remaining in the white matter retained the potential for amplification of pro-inflammatory signals that may account for the extend of tissue damage in Csf1-/- lesions.

Thus, we propose that Csf1-deficiency exerts an indirect effect on axonal survival by affecting the inflammatory reaction balance between activated microglia/macrophages and astrocytes which results in instigating the pro-inflammatory phase not followed by subsequent pro-regeneration phase of glial activity in course of demyelination/remyelination.

## 4. Discussion

Numerous CNS disorders are associated with microglia dysfunctions, therefore understanding the molecular mechanisms that regulate microglia homeostasis and function is important for their treatment [60,61]. Development and functions of microglia critically depend on colony-stimulating factor 1 receptor (Csf1R), the class III transmembrane receptor tyrosine kinase, a key regulator of myeloid cell biology [62,63,64,65,66] and its ligand Csf1 [67]. Our aim was, therefore, to decipher the consequences of Csf1 deficiency in white matter microglia in response to demyelination. We show that Csf1 deficiency increases axonal susceptibility to injury and impairs the efficiency of remyelination in focal white matter demyelinating lesions.

Previously incomplete microglia depletion in Csf1-deficient mice [68] contrary to profound ablation of microglia in Csf1R-deficient ones [63,64] where reported. However, we and others have shown severe reduction in microglial cell number in the spinal cord white matter but not in grey matter in Csf1-/- mice [69]. This discrepancy could be explained by functions of an alternate Csf1R ligand – IL-34 [70]. Both IL-34 and Csf1 are ligands for the same receptor, but they have distinct CNS distribution and cellular source [61,71]. IL-34 is produced by neurons and plays a vital role for microglial cells in the CNS grey matter, whereas astrocyte-derived Csf1 acts as the major ligand for the maintenance of white matter microglia [72]. Since Csf1R expression, the only receptor for Csf1, is prominent in microglia throughout the brain, Csf1R-/-mice have a more severe phenotype than the Csf1-/-, however, they exhibit increased neuronal density, elevated numbers of astrocytes but reduced numbers of oligodendrocytes and substantially poorer viability [67]. Numerous Csf1R inhibitors (e.g., PLX3397, PLX5622, and AFS98) have been developed as a novel strategy for experimental depletion of microglia in rodents [73,74], however, upon treatment cessation residual microglia rapidly repopulate the CNS [75].

Nevertheless, both heterogeneous expression pattern of Csf1R ligands as well as the consequences of the pathway disturbances have been identified in patients, where heterozygous mutations in the Csf1R have been linked to a severe neurodegenerative condition such as hereditary leukodystrophy with axonal spheroids [76], while homozygous mutations with a complete loss of microglia in the brain resulted in severe structural brain defects [77].

Given the above, to study Csf1 role in microglia function in remyelination we used the osteopetrotic mice, congenitally homozygous for a null mutation in the Csf1 gene, that have severely reduced number of microglia preferentially within spinal cord white matter [34,35]. What is important, neither development of axons (in terms of their density and diameter) nor developmental myelination (myelin thickness and ultrastructural integrity) was affected in Csf1-/- mice, which is consistent with the previously reported observations [69]. In the intact mutants, we found a profoundly reduced number of white matter microglia, while astrocyte number was unaffected. Since Csf1-/- mice have very few macrophages in the peritoneal cavity and severely reduced numbers of monocytes in the peripheral blood [78], this is unlikely that they participate in CNS inflammation. However, in response to CNS injury cells of the hematopoietic origin in the various differentiation stages can reach the lesion and contribute to macrophage populations. Several studies on chimera/transgenic mice have shown that bone marrow borne cells, including monocyte lineage cells and their progenitors, enter CNS and gradually undergo a dynamic functional transformation under the influence of CNS microenvironment. For example, GFP-transfected bone marrow precursors have been reported to repopulate the brain parenchyma with ramified morphology and co-expression of bona fide macrophage markers Iba-1 and CD11c [79]. Our observations suggest that Csf1 is responsible for both monocyte lineage cell proliferation and differentiation/maturation in astroglia-created environment, while Csf2 induces massive cell proliferation. We concluded therefore that Csf1-/- mice make a reliable and relevant tool to investigate the white matter pathophysiology in the microglia-deficient environment that is achieved regardless of animal age and without any pharmacological treatments or further genetic manipulations.

Toxin-induced demyelination triggers little damage to axons that spontaneously remyelinate after three weeks. Although this model is considered as a non-inflammatory, it has been shown that cytokines such as MCP1, MIP1a, Csf1/2, and TNFα are secreted and attract peripheral macrophages that contribute to lesion resolution [80]. These make the toxin model particularly useful to study the innate immune control of the remyelination.

We found that in Csf1-deficient mice microglia were not able to proliferate within the demyelinating lesions to the same extent as in WT mice. Similarly, previous studies have demonstrated that the proliferation of microglia is impaired in the Csf1-/- mice after cerebral ischemia [37], facial nerve axotomy [36], and intrahippocampal injection of kinate [38]. Although microglia in Csf1-/- mice are unable to proliferate, they can be activated under those pathophysiological conditions. For example, Rogove et al. [38] demonstrated that activation of as little as 40% of the microglia was sufficient to trigger neurodegeneration in kainic acid-induced neural injury. However, we show that the limited proliferative capability of microglia in Csf1-/- mice is associated with the dysregulation of their activation and further function of existing cells, which in consequence, significantly disturbs CNS white matter remyelination.

Although microglia have been reported to play a beneficial role in remyelination [20,21,27], the significant reduction in microglia number (around 86% in the spinal cord white matter; Figure 1) did not affect normal myelin development in the Csf1-/- mice. This supports the notion that proper microglia function might be important for effective remyelination rather than for normal myelin development by regulation of inflammation within injured tissue (reviewed in [81]). The influence of microglia activation on OPCs differentiation and in consequence, white matter repair after demyelination has been already extensively reported [82,83,84]. Removal of myelin debris that contains inhibitors of OPCs differentiation has been suggested as an essential mechanism ameliorating remyelination [27,28,82]. In our experimental model however, we found as many differentiated oligodendrocytes within previously demyelinated areas in Csf1-/- mice as in WT which suggests that low efficiency of remyelination observed in Csf1-/- mice might be a result of early axonal damage that occurred in demyelinated white matter rather than inhibition of OPC differentiation.

The growing evidence indicates that the immune response both contributing to the injury development at the early stages and supporting white matter repair and requires to be timely and properly regulated [18,85]. While the pro-inflammatory microglia/macrophages predominate in the lesion early after demyelinating insult, a gradual switch from an M1- to an M2-dominant phenotype occurs at the initiation of remyelination. The existing data clearly show that the early microglial activation in response to demyelination proceeds through at least two distinct temporal stages, which were defined by specific expression profile of inflammatory-associated genes [86]. At the very early stage, the vast majority of microglia/macrophages express the M1 marker inducible nitric oxide synthase (iNOS) than the M2 marker arginase 1 (Arg1). However, at the beginning of OPC differentiation/remyelination stage, there are significantly more Arg1-positive M2 cells than iNOS-producing M1 cells. This polarization switch has been confirmed as a critical factor for successful OPCs differentiation and remyelination [31]. Similarly, in EAE, cells of the regenerative phenotype were clearly evidenced during the remission phase [87]. Importantly, this functional shift can also be observed in MS patient lesions, as shown by identification of myelin-laden macrophages expressing high levels of pro-regenerative phenotype-associated markers [88], in inactive lesion centers, while iNOS expression was associated with areas of active pathology at lesion edges [89].

Although previous studies have highlighted the regenerative capacity of activated microglia/macrophages in effective remyelination, we now point to the importance of proper regulation of the pro-inflammatory phase of their response in this process. In this study, we provide evidence that Csf1 deficiency significantly disturbs expression of genes encoding for the proteins that have been proposed to be critically involved in M1/M2 switch. Such dysregulation substantially changes the lesion environment into a prolonged inflammatory condition resulting in remyelination failure. This could be either because the axons remain demyelinated and are vulnerable to atrophy or because they are exposed to severe inflammatory conditions before OPC differentiation despite the limited number of inflammatory microglia present in the lesion.

We show that in the absence of pro-inflammatory activated microglia, astrocytes take over their role and produce inflammatory factors at least longer than in wild type animals, which disturbs the timing of the microenvironmental inflammatory/regenerative switch. This results in inflammation-associated axonal transport disturbances (identified by detection of SMI32-positive axons) and axon disintegration which ultimately impedes complete remyelination. Thus, our results suggest that reduced remyelination in Csf1-/- mice per se is not solely responsible for the increased axonal loss, as the SMI32-positive axons were identified as early as at the initiation phase of OPC differentiation. This is consistent with the accumulating evidence that Csf1-/- axons may be intrinsically more susceptible to inflammatory-associated injury, for example, the observed vulnerability of Csf1-/- cortical neurons to an ischemic insult [37]. Our findings suggest an existence of a functional inflammatory cross-interaction: the altered pro-inflammatory activation of microglia/macrophages in Csf1-/- mice causes a strong aberrant activation of astrocytes which significantly contributes to further axonal damage. This is of biological relevance as transected axons are common features of the lesions of multiple sclerosis. Their frequency is correlated to the degree of inflammation within the lesion and the irreversible neurologic impairment [90], and makes a significant contribution to the progressive phase of MS [91].

While it is well documented that the myelin sheath is important for the survival of axons by providing physical and metabolic support in the developing CNS [92,93,94], accelerated remyelination has been also shown to support axonal integrity and restoring neuronal function in an inflammatory condition like MS [95]. Selective depletion of inflammatory monocytes [96] as well as genetic microglial inactivation in transgenic mice [97] reduces the severity of EAE, the murine model of MS. Similarly, microglia ablation by administration of Csf1R inhibitor significantly reduced demyelination and immune activation which resulted in attenuating EAE [98] and in chronic demyelination model [99]. Moreover, it has been reported that Csf1 upregulation may play an important role in the proliferation and activation of microglia in EAE, contributing to neuroinflammation and neurodegeneration. Viral vector mediated over-expression of Csf1 in spinal neurons induced profound proliferation and activation of microglia and subsequent significant loss of CSF1-transduced neurons and demyelination in the Csf1-transduced areas [100]. On the other hand, the pharmacological depletion of microglia in a model of focal demyelination led to delayed OPCs recruitment, impaired myelin protein synthesis, and as a result reduced remyelination [22,26,27]. Moreover, the pro-regenerative phenotype of microglia/macrophages encourages OPC differentiation [86]. The differences in the functional outcome of microglia deficiency between EAE and a remyelination model reported here likely originate from the nature of the immune response in course of experimental toxin-induced demyelination and a peripherally driven neuroinflammation model such as EAE. EAE-evoked white matter lesions in microglia/macrophage-depleted animals showed substantial preservation of mature, myelinating oligodendrocytes in comparison to control animals. This suggests that the ablation of microglia/macrophages during the symptomatic phase of EAE reduces inflammation and promotes recovery. This could be explained by the fact that inflammatory demyelination in course of EAE is accompanied by significant neuronal loss and the continued progression of the disease is largely due to their role in monocyte recruitment. Endogenous remyelination in this model is influenced by ongoing inflammation and neurodegeneration, and it is nearly absent at the peak of EAE and an inefficient at the later stages.

## 5. Conclusions

Emerging evidence couples the successful reconstruction of demyelinated white matter with activation of the pro-regenerative phenotype of microglia/macrophages. Therefore, it has been widely postulated that modulating microglia activation to minimize the pro-inflammatory and favor the regenerative polarization could be a beneficial and promising strategy in future treatments of diseases with demyelination components. Our results contribute to the general view that inflammation is a dynamic process that proceeds in consecutive, timely, and tightly regulated stages, with an early pro-inflammatory phase followed by regeneration and restoration of tissue homeostasis. However, we clearly showed that deficiency of inflammatory microglia/macrophages results in dysregulation of kinetics and a regulatory balance of immune responses within demyelinated white matter. This, in consequence, increases vulnerability to demyelination and inhibits a regenerative outcome. As outlined, we identified Csf1 as the essential factor that regulates microglia orchestration of the pan-glia interactions that support the myelin sheath reconstruction.
